# The Effect of Implicitly Incentivized Faking on Explicit and Implicit Measures of Doping Attitude: When Athletes Want to Pretend an Even More Negative Attitude to Doping

**DOI:** 10.1371/journal.pone.0118507

**Published:** 2015-04-22

**Authors:** Wanja Wolff, Sebastian Schindler, Ralf Brand

**Affiliations:** 1 Division of Sport and Exercise Psychology, University Potsdam, Potsdam, Germany; 2 Department of Psychology, University of Bielefeld, Bielefeld, Germany; 3 Center of Excellence Cognitive Interaction Technology (CITEC), University of Bielefeld, Bielefeld, Germany; University of Rome, ITALY

## Abstract

The Implicit Association Test (IAT) aims to measure participants’ automatic evaluation of an attitude object and is useful especially for the measurement of attitudes related to socially sensitive subjects, e.g. doping in sports. Several studies indicate that IAT scores can be faked on instruction. But fully or semi-instructed research scenarios might not properly reflect what happens in more realistic situations, when participants secretly decide to try faking the test. The present study is the first to investigate IAT faking when there is only an implicit incentive to do so. Sixty-five athletes (22.83 years ± 2.45; 25 women) were randomly assigned to an incentive-to-fake condition or a control condition. Participants in the incentive-to-fake condition were manipulated to believe that athletes with lenient doping attitudes would be referred to a tedious 45-minute anti-doping program. Attitudes were measured with the pictorial doping brief IAT (BIAT) and with the Performance Enhancement Attitude Scale (PEAS). A one-way MANOVA revealed significant differences between conditions after the manipulation in PEAS scores, but not in the doping BIAT. In the light of our hypothesis this suggests that participants successfully faked an exceedingly negative attitude to doping when completing the PEAS, but were unsuccessful in doing so on the reaction time-based test. This study assessed BIAT faking in a setting that aimed to resemble a situation in which participants want to hide their attempts to cheat. The two measures of attitude were differentially affected by the implicit incentive. Our findings provide evidence that the pictorial doping BIAT is relatively robust against spontaneous and naïve faking attempts. (B)IATs might be less prone to faking than implied by previous studies.

## Introduction

Assessment of attitudes on socially sensitive subjects using traditional self-report measures is susceptible to faking [1,2]. The Implicit Association Test (IAT) aims to reflect test participants’ automatic evaluations of an attitude object. It is a reaction time-based, computerized sorting task intended to conceal the true measurement target, and thus potentially more resistant to faking [3–5]. Research has shown that IATs are especially useful in the assessment of attitudes related to socially sensitive subjects (e.g., stereotyping or prejudice) [6]. Faking an IAT on the very first exposure appears to be almost impossible [1,2,7], but evidence is accumulating that IATs can be faked to some extent when participants are allowed to become familiar with the test format [7,8].

All previous studies have explicitly asked participants to try faking their IAT score [2,7–10]. Successful faking has been assessed using both within- and between-subject designs [2,10], and in some studies participants were given explicit instructions on how to fake the test (e.g., slowing of responses in sensitive trials and reacting as quickly as possible in others) [7,8]. In summary, extant studies indicate that successful IAT faking increases when participants are given repeated opportunities to fake the test, and that provision of an effective faking strategy is an even more effective aid to faking [2,9].

Instructing participants to fake test scores in order to assess the vulnerability of the test to faking, or to learn how to detect faking attempts, is a method which has also been used in other research areas e.g. in memory malingering [11,12]. In these studies participants are usually assigned to a faking or a non-faking condition by the experimenter. This approach secures internal control on the participants’ group membership [13]. Other studies from the same research area however suggest that participants who have been instructed to fake poor performance on memory tests tend to over-exaggerate their memory deficits [12,14].

Taking our cue from this line of research it is suggested that the same could happen when participants are asked and instructed to fake an IAT score: results from instructed faking attempts (a behavior that is ethically acceptable in this case) might differ from uninstructed, hidden faking attempts. We believe that this difference might account for conflicting data on the IAT’s vulnerability to faking [7,15] as well as for ambiguous findings with respect to the IAT’s predictive validity [5].

As indicated by other research from memory malingering, it is difficult to determine whether people who are involved in litigation are faking or not. The presence of an objective, external incentive for faking is one important criterion for classifying a claimant’s performance as fraudulent [16]. However, with regard to our study on IAT faking, an experimental manipulations should be used that provides participants with an implicit incentive to hide their true attitudes. They should be incentivized to alter their responses in order to achieve or avoid a certain consequence. Faking motivated in this way is likely to differ from faking elicited by a researcher’s request. As cheating on a test is regarded a socially condemned behavior, participants should believe that their attempt to fake has a fair chance to remain undetected.

Athletes’ attitudes to doping are subject to social pressure in a way that they are expected to be strongly anti-doping. Direct assessments with attitude questionnaires are heavily biased by socially desirable responding [17]. Attitudes to doping are therefore a suitable substrate in which to investigate the IAT’s susceptibility to faking. In the following study we tested the hypothesis that in an experimental condition in which an implicit incentive to fake was present, participants would be capable of faking an attitude on a questionnaire, the Performance Enhancement Attitude Scale (PEAS) [18], but not on a response time-based measure, the doping Brief IAT (BIAT) [19].

## Method

### Participants

Our sample consisted of 65 university sports students (mean age: 22.83 years ± 2.45 years; 25 women) who participated in return for course credit. All reported that they competed regularly in sports tournaments or leagues, and it is reasonable to assume that all of them had formed attitudes to doping either as a result of participating in competitions, or on the basis of their university studies.

### Procedure and Treatment

Participants were randomly allocated to either an implicit incentive-to-fake (*n* = 30) or to a no-treatment control condition (*n* = 35). All of them were told that they would be participating in a test validation study and that the experiment was about finding out whether two tests of attitude to doping (the PEAS and the doping BIAT) would produce comparable results.

All participants began with a doping BIAT (see below) practice trial in order to familiarize themselves with this test and to increase the probability that they would be able to fake it (as even instructed faking has been shown to be almost impossible upon the very first exposure to the IAT) [7]. This practice trial was described as part of the standard procedure in IAT-based attitude testing. Participants were then randomly assigned to the incentive-to-fake or control condition.

In the incentive-to-fake condition participants were informed that results from both tests, the doping BIAT as well as the questionnaire, would also be used to detect unacceptably lenient attitudes to doping and that participants whose doping attitudes are “too positive” would be required to attend a tedious, automated anti-doping lecture immediately after the attitude tests, whereas participants whose test performance indicated an appropriately negative attitude to doping would be free to leave the laboratory immediately after completing the attitude tests. The explanation given for this was that it was university policy to discourage lenient attitudes to doping, especially among sports students.

The experiment’s incentive to fake is that faked scores might lead to a favorable outcome for the participant (i.e. avoid the anti-doping lecture). The incentive is implicit in a way that it does not explicitly encourage participants to try faking; it rather intends to make faking a tempting behavioral option. All instructions were intended to ensure that participants would view scores indicating a negative attitude to doping on both tests as the desirable outcome. In the control condition participants were simply asked to complete the two tests and were reassured that the direction of their attitude scores would not be evaluated at all.

All participants completed the response time-based test before the questionnaire [6]. Then socio-demographic data and data for the manipulation check were collected. Participants were fully debriefed and thanked for their participation at the end of the experiment.

Experimental deception was in line with standard 8.07 of the American Psychological Association (APA) Ethical Principles of Psychologists and Code of Conduct [20]. Verbal informed consent to participate in the experiment was documented anonymously in the study protocol by the research assistant beforehand, thereby following the German Psychological Society’s (DGP) guidelines (C.III. paragraph 6; recommended when data acquisition is anonymous and it is expected that the experiment does not induce any harm or more discomfiture than in everyday life).

### Measures

Data collection was fully computerized. The Inquisit 3.0 software (http://www.millisecond.com/) was used to run the doping BIAT and present the PEAS.

### BIAT

The BIAT is a shorter variant of the standard IAT procedure [21]. The pictorial doping BIAT is a validated, indirect test for measuring athletes’ automatic evaluations of doping [19,22]. The test begins with a training block (20 trials), in which participants learn to discriminate between focal doping and non-focal health food pictures. This is followed by one *doping* + *like* block (20 trials) and one *doping* + *dislike* block (20 trials). The order of the two test blocks is randomized across participants to control for position effects. D-scores were calculated according to the improved scoring algorithm [23] such that more positive scores indicate a more lenient attitude to doping.

### The PEAS

The PEAS is a seventeen-item questionnaire which measures athletes’ evaluative judgments of doping [18]. Participants are required to indicate their agreement or disagreement with a series of statements, using a six-point Likert-type scale from 1 = “strongly disagree” to 6 = “strongly agree”. Sample statements are “Legalizing performance enhancements would be beneficial for sports”, “Athletes are pressured to take performance-enhancing drugs” and “Only the quality of performance should matter, not the way athletes achieve it”. PEAS scores can range between 17 and 102 (with a theoretical mean of 59.5). Higher scores indicate a more lenient doping attitude. A validated German translation of the PEAS was used for the present study [19].

### Control variables

A validated German version of the Balanced Inventory of Desirable Responding [24] was used to control for inter-individual differences in socially desirable responding. This scale measures two facets of social desirability *self-deceptive enhancement* and *impression management*. Both facets are assessed on the basis of responses to ten statements, given on a seven-point Likert-type scale ranging from 1 = “complete refusal” to 7 = “complete affirmation”. Scores for each subscale can range between 10 and 70 (with a theoretical mean of 40). In this study Cronbach’s alpha was. 48 for the self-deceptive enhancement subscale and. 68 for the impression management subscale. We did not include data from the self-deceptive enhancement subscale in subsequent analyses owing to the very low reliability it exhibited.

### Manipulation check

Participants were asked two dichotomous (yes vs. no) questions, “Did you try to fake the BIAT (reaction time measure)?” and “Did you try to fake the PEAS (questionnaire)?” in order to reveal any participants who were prepared to state explicitly that they had tried to fake their test results. Willingness to admit to faking would indicate that our implicit incentive to fake had failed, in the sense that it would indicate that participants did not feel that being caught faking would have negative consequences. A third question assessed recollection of the name of the bogus anti-doping program as a check of awareness of the consequences of being found to have an overly positive attitude to doping.

### Statistical Analyses

An unpaired t-test was calculated in order to check for inter-individual differences (group differences) in social desirability. A one-way (incentive-to-fake vs. no treatment) multivariate analysis of variance (MANOVA) was used to determine whether the experimental manipulation led to differences in scores on the doping BIAT or the PEAS.

## Results

### Descriptive Statistics

Descriptive statistics for the PEAS, BIAT and the impression management scale are given in Table 1. Two participants admitted to having tried faking the PEAS. All participants denied trying to fake the doping BIAT. Two-third (66.67%) of participants in the incentive-to-fake condition correctly remembered the name of the anti-doping program, indicating that most were aware of the bogus training program. An independent samples *t*-test showed that participants in both conditions had similar dispositions of socially desirable responding, *t*(63) = 0.24, *p* =. 81, so that this variable was not added as a covariate to the subsequent MANOVA.

**Table 1:** Descriptive statistics for attitudes and strategic responding in groups.

	PEAS^a^		BIAT^a^		Impression management^b^	
Condition	*M (SD)*	*Min-Max*	*M (SD)*	*Min-Max*	*M (SD)*	*Min-Max*
Control	30.74 (6.96)	17–47	-0.37 (0.46)	-1.35-+0.63	46.97 (10.93)	23–69
Incentive-to-fake	26.63 (6.26)	17–44	-0.27 (0.52)	-1.13-+0.79	46.33 (10.15)	25–65

^a^Low test scores represent negative doping attitudes;

^b^Low test scores indicate low dispositions.

### Main Analysis

**Fig 1:**
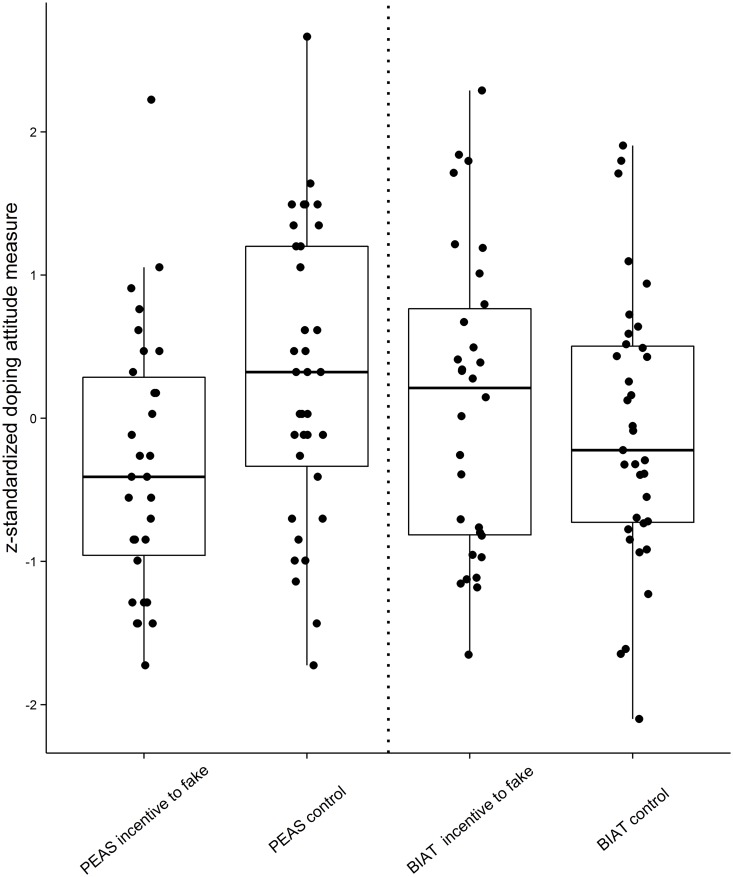
Z-standardized scores (boxplots with data point distributions) from the response-time based (BIAT) and questionnaire (PEAS) doping attitude measures in the two experimental conditions.

The MANOVA revealed a significant omnibus effect with medium effect size, *F*(2, 62) = 3.72, *p* =. 05, η^2^ =. 10. Subsequent univariate tests showed that the PEAS scores, *F*(1, 63) = 6.17, *p* =. 02, η^2^ =. 09, but not the doping BIAT scores, *F*(1, 63) = 0.57, *p* =. 45, η^2^ =. 01, were significantly lower in the incentive-to-fake group than in the control group. Standardized mean scores for the PEAS and the doping BIAT are presented as a function of experimental condition (i.e. in the two groups) in Fig 1.

## Discussion

This is the first study to investigate BIAT faking using an implicit incentive-to-fake condition instead of explicitly instructing participants to try faking the test. In our experiment half of the participants were informed that detection of an unacceptably lenient doping attitude would lead to an additional arduous subsequent task. These participants then displayed significantly more negative evaluations of doping in a doping attitude questionnaire than control participants in a no-treatment condition. The experimental manipulation did not produce similar differences in our participants’ attitudes. On the pictorial doping BIAT, participants given an implicit incentive to fake, displayed attitudes to doping similar to those of control participants without the implicit incentive to fake. These effects can be attributed to our experimental manipulation since assignment to conditions was random.

Because of the subtle manipulation we used we are unable to determine whether or not participants actually tried to fake their tests. It is possible that our participants were able but unwilling to fake the doping BIAT or that they were willing but unable. Fear of being caught could have decreased our participants’ motivation to fake their tests. But, most crucially, no matter whether participants were unable or unwilling to fake, only the response-time based attitude scores showed up to be unaffected by the experimental treatment. This pattern of results is similar to results from a study by Petróczi and colleagues [25] who found that those assumed to lie about their doping behavior (based on discrepancy between self-reported absence from doping and hair analysis) produced extreme negative test results across all questionnaire measures (PEAS, projected prevalence of doping, perceived pressure to dope, etc.) but not so much on the IAT. With regard to our study, the hypothesized and finally observed group difference on the questionnaire suggests that faking occurred. Since participants cannot be expected to truthfully answer whether or not they tried to fake any of the two tests, there is no way of determining post-hoc whether or not participants tried to alter their responses on the BIAT. It could be argued that participants were motivated to alter their responses only on the PEAS; however, research on memory malingering has demonstrated that claimants producing suspicious results on one symptom validity test also tend to show suspicious performance on other validity tests and cognitive measures [26]. Further research is needed to disentangle differential motivation-to-fake vs. capacity-to-fake effects though.

All participants were informed that both the pictorial doping BIAT (the response time based attitude test) and the PEAS (the attitude questionnaire) were accurate measures of attitudes to doping. This deception was necessary for the experimental manipulation to work although both measures are far from perfect measures and although one should not use these tests for individual diagnostics in fact. Hiding the measurement target has been regarded as one reason for the relative robustness of indirect measures against faking in previous studies [4,27]. In our study however, knowing what latent construct the BIAT test supposedly measured, did not result in successful faking. It seems that, at least under a subtle incentive to fake, it is not *what* the BIAT measures but *how* it is measured that is responsible for its robustness against faking. Implicitly incentivized faking attempts might lower the predictive validity of direct measures of attitudes related to socially sensitive subjects but, according to our data, indirect tests that aim to measure participants’ automatic evaluations are unlikely to be affected.

Most of the extant findings on IAT faking are based on ‘instructed faking’ paradigms with participants being supplied with explicit guidance on how to fake. Research on memory malingering has shown that participants who are instructed to malinger tend to perform differently from claimants who are suspected of malingering [12,14]. The same may hold true for attitude testing, especially when participants work on response time-based indirect tests. Our aim was to establish and use an implicit ‘incentive-to-fake’ paradigm making it possible to investigate the possibility of faking and faking effects more realistically. Our results suggest that (B)IATs could be less prone to faking than suggested by earlier research. The IAT’s vulnerability to faking might has been overestimated so far; provided that other researchers are able to replicate our finding.

In most previous experiments, unlike in real life settings, there was no reason for participants to fear the consequences of faking being detected. Faking in real world situations is therefore likely to be more subtle, since fakers do not want to get caught and do not necessarily regard their performance as faking in the first place. Telling participants that it was possible to detect faking would have enhanced the external validity of the paradigm; the manipulation would have provided both a situational incentive to fake and a disincentive (threat of punishment) for detectable faking then. Future studies should investigate how response patterns change when rewards and punishment for faking are manipulated in conjunction. Participants may adopt a different faking strategy when they believe that faking might be detected, but they are also less likely to make the attempt [28].

### Limitations

We used a between-subjects design. A within-subjects assessment of changes in the attitude questionnaire and in the response time-based test following a faking manipulation would have been preferable. However this was ruled out by the nature of our experimental manipulation, which did not permit the use of a counterbalanced within-subjects design. The threat of the bogus anti-doping training in the experimental condition, could not reasonably have been abolished when the control condition occurred after the experimental condition. Using a non-counterbalanced presentation of experimental and control conditions would have been problematic as IAT performance is influenced by practice. We therefore believe that the chosen between-subjects design represented the best approach to addressing our research question.

The results presented here pertain to one specific BIAT, namely the pictorial doping Brief IAT [19,22]. Generalizations from our results to, for example, other IAT types should be made with caution. The Single Category IAT (SC-IAT) for instance, an IAT variant with only one target and no comparison concepts [29], has been found to be more prone to faking than standard IAT’s [10]. The SC-IAT might be more malleable to faking in the implicit incentive-to-fake paradigm as well. Further one cannot rule out that it is easier to fake attitudes in certain domains or towards certain types of stimuli. We propose that future research should use implicit incentive-to-fake paradigms to investigate possible differences in malleability towards faking as a function of the test stimuli used (e.g., pictures vs. words), the type of the IAT (e.g., SC-IAT vs. BIAT) and the attitude object (e.g., doping vs. stereotypes about race).

## Conclusion

This study provides first evidence that BIAT faking (and presumably also IAT faking more generally) is less likely than implied by the extant literature. A situational manipulation that was sufficient to alter participants’ responses on an attitude questionnaire did not lead to alterations in scores on a reaction time-based measure of attitude to doping. In our view, this finding supports the claim that the BIAT is difficult to fake [27]. Valid, robust tests are a prerequisite of social science research directed at understanding and preventing doping. While it has to be acknowledged that a multitude of situational and personal factors affect doping behavior [30], indirect measures such as the (B)IAT hold promise in explaining incremental variance in doping behavior [25]; the more so as research in doping-related automatic cognition is still in its infancy [31]. The pictorial doping BIAT used here has already been shown to statistically significant discriminate between positive and negative biochemical doping test results [22]. It is important to note however that results from indirect tests intend to tap automatic human thinking processes and should not be seen as “doping tests”. Evidence of a response time-based indirect attitude test’s robustness in a realistic faking scenario supports its potential utility as a valid research instrument that helps to deepen our understanding of doping-related cognition and—as a consequence—to develop (even) more effective doping prevention interventions.

## Supporting Information

S1 Dataset sex = participants gender (1) female (0) male. subject = given subject number. Bedingung = condition (1 = incentivized to fake, 0 = control condition). t1 = BIAT practice trial. MA = doping+like block MB = doping+dislike block. d1 = D-score. Order = start with doping+like (1) or doping+dislike (2) first. t2 = BIAT test trial. PEASxy = PEAS scale questionnaire items Faking BIAT = admitted to faking on the BIAT (1) or not (0). Faking PEAS = admitted to faking on the PEAS (1) or not (0). Faking success = estimation when faking was admitted about faking success. SozErwxy / SozExy = BIDR questionnaire items of desirability responding. SozErw_umPoolxy / SozE_Rxy = recoded items age = participants age. sex = participants gender. sport = named sport in which participants were or are competing with others. NameEthikRecog = recognition of the name of the tedious anti doping program (1) or not (0). InhaltEthikRecog = name of the program. SozErw_ST_Sum / SozErw_FT_Sum = BIDR score for the scales impression management and self-deceptive enhancement. PEAS = score for the PEAS questionnaire ZPEAS = standardized PEAS score. Z_DScore_umk = changed sign for the standardized test trial D-score. Z_t2_d1 = standardized test trial D-score.
(ZIP)
