# Supplementary material for: The Effect of Implicitly Incentivized Faking on Explicit and Implicit Measures of Doping Attitude: When Athletes Want to Pretend an Even More Negative Attitude to Doping
Source: PLoS One. 2015 Apr 22;10(4):e0118507. doi: 10.1371/journal.pone.0118507 (PMC4406708; doi:10.1371/journal.pone.0118507)
Supplement: S1 Dataset — subject = given subject number. Bedingung = condition (1 = incentivized to fake, 0 = control condition). t1 = BIAT practice trial. MA = doping+like block MB = doping+dislike block. d1 = D-score. Order = start with doping+like (1) or doping+dislike (2) first. t2 = BIAT test trial. PEASxy = PEAS scale questionnaire items Faking BIAT = admitted to faking on the BIAT (1) or not (0). Faking PEAS = admitted to faking on the PEAS (1) or not (0). Faking success = estimation when faking was admitted about faking success. SozErwxy / SozExy = BIDR questionnaire items of desirability responding. SozErw_umPoolxy / SozE_Rxy = recoded items age = participants age. sex = participants gender. sport = named sport in which participants were or are competing with others. NameEthikRecog = recognition of the name of the tedious anti doping program (1) or not (0). InhaltEthikRecog = name of the program. SozErw_ST_Sum / SozErw_FT_Sum = BIDR score for the scales impression management and self-deceptive enhancement. PEAS = score for the PEAS questionnaire ZPEAS = standardized PEAS score. Z_DScore_umk = changed sign for the standardized test trial D-score. Z_t2_d1 = standardized test trial D-score. (ZIP) [file pone.0118507.s001.zip › S1_Dataset legend.docx]

Legend to the data file Wolff et al. 2014:

subject = given subject number

Bedingung = condition (1=incentivized to fake, 0=control condition)

t1 = BIAT practice trial

MA = doping+like block

MB = doping+dislike block

d1 = D-score

Order = start with doping+like (1) or doping+dislike (2) first

t2 = BIAT test trial

PEASxy= PEAS scale questionnaire items

Faking BIAT = admitted to faking on the BIAT (1) or not (0)

Faking PEAS = admitted to faking on the PEAS (1) or not (0)

Faking success = estimation when faking was admitted about faking success

SozErwxy / SozExy = BIDR questionnaire items of desirability responding

SozErw_umPoolxy / SozE_Rxy = recoded items

age = participants age

sex = participants gender

sport = named sport in which participants were or are competing with others

NameEthikRecog = recognition of the name of the tedious anti doping program (1) or not (0)

InhaltEthikRecog = name of the program

SozErw_ST_Sum / SozErw_FT_Sum = BIDR score for the scales impression management and self-deceptive enhancement

PEAS = score for the PEAS questionnaire

ZPEAS = standardized PEAS score

Z_DScore_umk = changed sign for the standardized test trial D-score

Z_t2_d1 = standardized test trial D-score
